# Initial Plant Vigor and Short Rotation Coppices Improve Vegetable Production in *Vitex doniana* Sweet (Lamiaceae)

**DOI:** 10.3390/plants9101253

**Published:** 2020-09-23

**Authors:** Sognigbe N’Danikou, Aristide Carlos Houdegbe, Dedeou Apocalypse Tchokponhoue, Aboegnonhou Odette Chaldia Agossou, Françoise Assogba Komlan, Raymond Sognon Vodouhe, Adam Ahanchede, Enoch Gbenato Achigan-Dako

**Affiliations:** 1 Laboratory of Genetics, Biotechnology and Seed Science, University of Abomey-Calavi, Abomey-Calavi BP 2549, Benin; houdariscarl@gmail.com (A.C.H); dedeoutchokponhoue@gmail.com (D.A.T.); achaldia.aboegnonhou@gmail.com (A.O.C.A.); rvodouhe@gmail.com (R.S.V.); ahanchedeadam@yahoo.fr (A.A.); e.adako@gmail.com (E.G.A.-D.); 2 École d’Horticulture et d’Aménagement des Espaces Verts, Université Nationale d’Agriculture, Kétou BP 43, Benin; 3 World Vegetable Center, East and Southern Africa, Duluti P.O. Box 10, Arusha, Tanzania; 4 National Agricultural Research Institute of Benin (INRAB), Cotonou BP 884, Benin; fasso2kom@gmail.com

**Keywords:** traditional vegetables, underutilized species, pruning, domestication, black plum

## Abstract

*Vitex doniana* is a major but threatened economic plant collected as a leafy vegetable and fruit in West Africa. How the species withstands coppicing as an agricultural management practice was investigated in this research. Three seedling vigor classes (10–25 mm, 25–30 mm, 30–40 mm) and two coppicing heights (20 and 40 cm) were compared to controls in eight replicates using a randomized complete block design. Mixed effect models were used to compare the effects of treatments on sprouting intensity, sprout growth, and biomass yield in the short (12 months) and medium term (three and five years). Results indicated that *V. doniana* is a fast-growing species, with heights between 2.72–7.73 m and diameter at breast height between 4.46–14.64 cm in five years. Vigorous (basal diameter > 30 mm) and shorter coppices (20 cm high) produced a higher number of sprouts. Although biomass yield was relatively higher on high coppices, the difference was not statistically significant. While a more severe harvesting regime was detrimental to plant growth, *V. doniana* can be managed to produce both vegetables and fuel wood in the medium term. These findings are significant for further improvement of the species, for food security, and climate resilience.

## 1. Introduction

*Vitex doniana* Sweet (a potential tree crop) is an example of wild-harvested species with a sophisticated market chain, which provides tangible incomes that support the livelihoods of many households in West Africa, particularly in Benin and Nigeria. Its fresh and tender leaves are perceived as a high quality vegetable in communities where it is consumed [1,2,3], and fruits are used for syrups and jams [4]. The wood of the species is reported to show similar technical characteristics as teak (*Tectona grandis* L.) [5], and can thus be used in furniture industries. However, the natural production of the species can no longer meet the ever-increasing demand and pressure on wild populations [1,2,6].

Domestication is suggested when there is an increased demand for natural products that wild-harvesting cannot meet. In other words, economic demand is thought to justify the need for domestication [7]. *Vitex doniana* is in such a situation and is listed as a priority tree crop that requires more research and adequate management [8]. While few studies have investigated regeneration of the species [9,10], there is still a need to explore the horticultural and silvicultural potentials, in order to develop agronomic packages for the cultivation and an increased availability.

The main product collected on *V. doniana* is its young leaves for vegetables. Therefore, the ability of the species to withstand branch and stem mutilation as harvesting practices is important in order to tailor agronomic techniques in horticultural systems. In fact, the success of any biomass plantation highly relies on seedling vigor and the ability of the species to survive coppicing and successive pruning systems [11,12]. A number of both intrinsic and external factors affect stand regeneration. These include plant vigor, coppice height, cutting season, cutting method, site quality, spacing, rotation length, provenance, genotype, and the species themselves [11,13]. Therefore, the study aimed at investigating how *V. doniana* responds to short rotation coppices as agricultural management practice and whether the initial stand vigor has any implications for the productivity of the managed plants for leafy vegetable production. The following hypotheses formed the basis for the study:

H1: The initial seedling vigor at transplanting is determinant for sprouting intensity in *V. doniana;*

H2: Lower coppicing height promotes regrowth and biomass yield in the species; and

H3: Coppicing as cultivation management to produce edible leaves negatively affect wood yield in the species.

## 2. Results

### 2.1. Survival of V. doniana to Transplanting and to Coppicing

**Figure 1:**
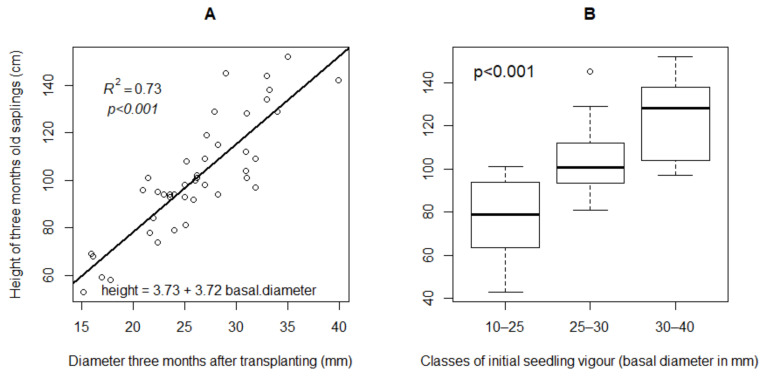
Growth of *V. doniana* saplings in height three months after transplanting. (**A**) Regression plot between plant diameter and height. (**B**) Boxplot of mean height per initial seedling vigor class. R^2^: Adjusted determination coefficient of the regression model; *p* < 0.001: correlation between height of sapling and basal diameter of sapling highly significant.

All *V. doniana* seedlings survived after transplanting and coppicing (*SRti* = 100% in all treatments). After three months and before experimental treatments were applied, the height of the saplings varied between 43 cm to 152 cm (Figure 1A). Three months after transplanting, and on average, more vigorous saplings with initial basal stem diameter (IBSD) > 30 mm grew faster in height compared with thinner ones (*p* < 0.05). The average height was 78.32 ± 18.04 cm for the thinner saplings (IBSD ≤ 25 cm), 104.50 ± 16.21 cm for medium size (25 > IBSD < 30 mm), and 122.38 ± 19.08 cm for vigorous saplings (IBSD ≥ 30 mm) (Figure 1B). Data also indicates a linear relationship (*y = ax + b*) between stem diameter and plant height in growing saplings, before the application of the management treatments (Figure 1A). The coefficient of determination (R^2^ = 0.73, *p* < 0.001) indicates a strong linear relationship between the stem diameter and plant height.

After five years, the survival rate was 100% in both the control trees and in trees that received experimental treatments. The data also showed that the diameter at breast height (DBH) of the control trees reached 11.67 cm (6.24 ± 2.60 cm on average) in three-year and 14.65 cm DBH (9.10 ± 3.01 cm on average) in five-year old trees. Control trees reached 4.14 m high on average (confidence interval CI: 2.18 m–5.89 m), and 5.36 m high on average (CI: 2.72 m–7.73 m) in three and five years, respectively.

### 2.2. Effects of Initial Vigor and Coppicing on the Sprouting Capacity and Biomass Production in the Short Term

#### 2.2.1. Number and Length of Shoots after Each Harvest

**Figure 2:**
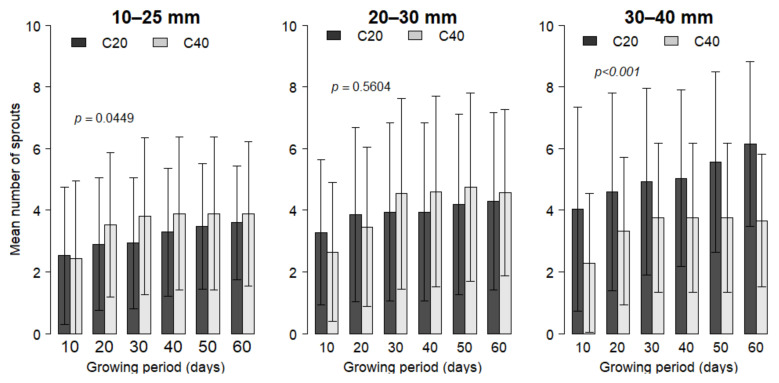
Number of sprout per initial plant vigor and per coppicing regime after each pruning, over 60 days after pruning. Four pruning series were applied. Coppicing regimes: C20 = coppicing at 20 cm above ground; C40 = coppicing at 40 cm above ground. Initial vigor classes: ISBD1 = 10–25 mm; ISBD2 = 20–30 mm, ISBD3 = 30–40 mm. *p*-values indicate the significance level of difference in sprouting among treatments. *p* > 0.05: No difference; *p* < 0.05: significant difference; *p* < 0.001: highly significant difference.

**Figure 3:**
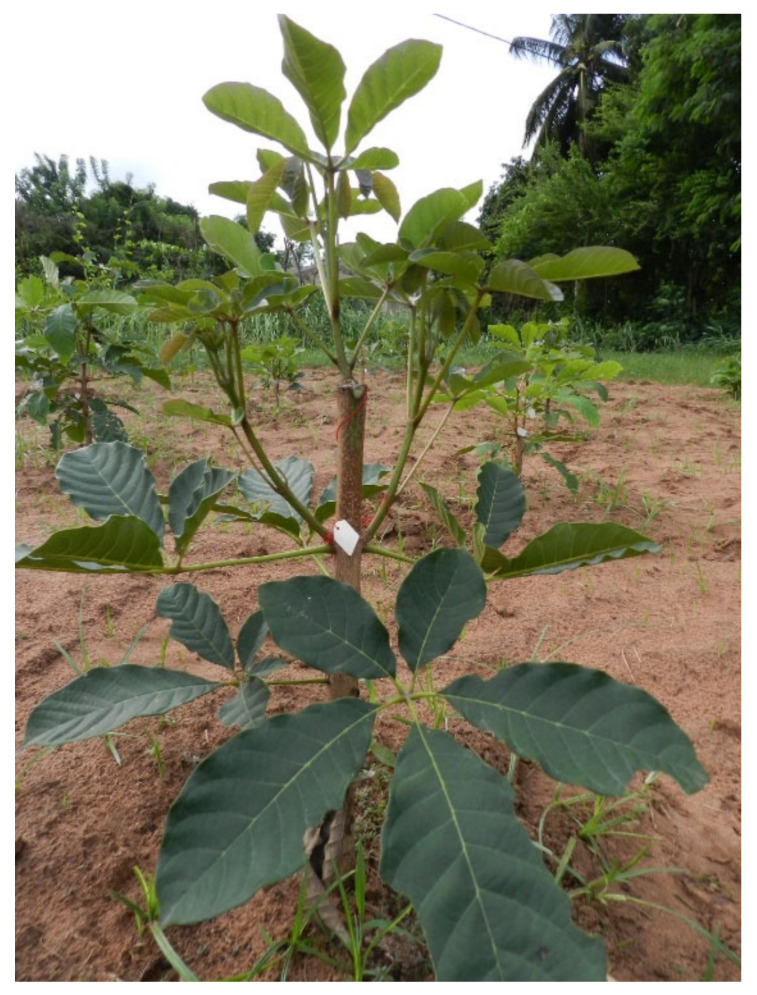
A sprouting high coppice of *V. doniana* in the experiment.

Results indicated that between two harvests, the number of new shoots increased (Figure 2). The number of shoots produced after each harvest varied between 0 and 12 (4.31 ± 2.55 sprouts on average). The more vigorous seedlings at transplanting (IBSD > 30 mm) showed higher sprouting capacity on short coppices at 20 cm above ground (C20), while the opposite trend was noticed on thinner seedlings at transplanting (IBSD ≤ 25 mm) with taller coppices exhibiting higher sprouting capacity. Figure 3 presents a sprouting high coppice of *V. doniana*. The variance analysis indicated highly significant effects of initial seedling vigor at transplanting (*p* < 0.001) and a marginal effect of coppicing height (*p* = 0.067) on the number of sprouts. There was a highly significant interaction effect of initial seedling vigor and coppicing height on the sprouting capacity (*p* < 0.001), and the best sprouting was recorded on shorter coppices (C20) that grew from initially vigorous seedlings (IBSD > 30 mm).

**Figure 4:**
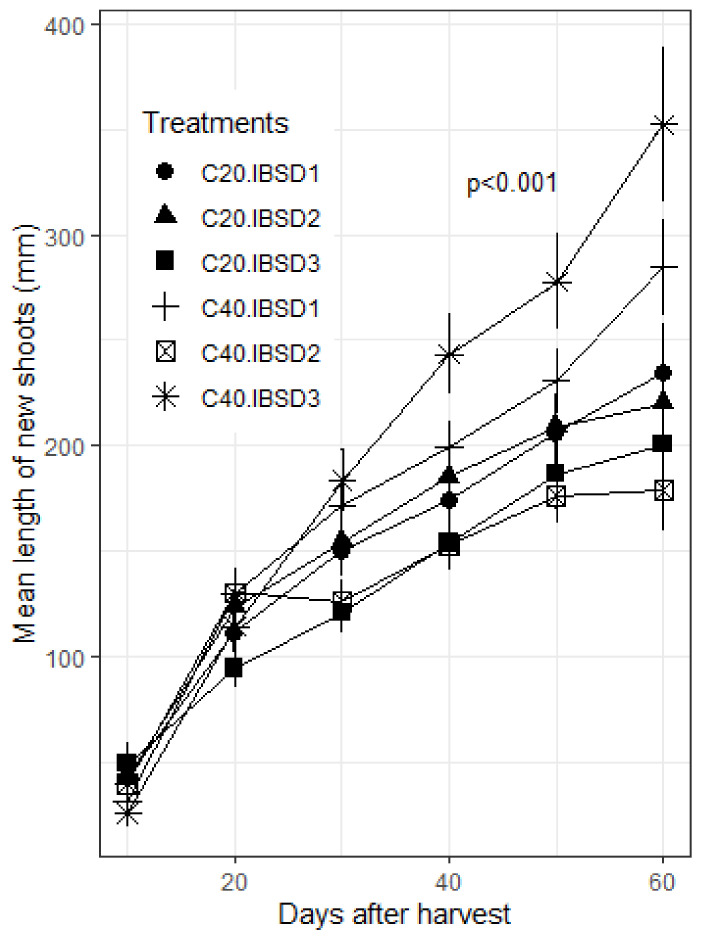
Length of new shoots per treatment, over 60 days after pruning. Coppicing regimes: C20 = coppicing at 20 cm above ground; C40 = coppicing at 40 cm above ground. Initial vigor classes: ISBD1 = 10–25 mm; ISBD2 = 20–30 mm, ISBD3 = 30–40 mm. *p* < 0.001: highly significant difference in length of new shoots among treatments.

Within 60 days, the length of new shoots varied from 0.40 cm to 104.80 cm, when considering all the applied management treatments (Figure 4). On average, sprouts length on the 60th day was 217.47 ± 12.40 mm and 262.36 ± 15.02 mm on the shorter and the longer coppices, respectively. On the small, medium, and vigorous seedlings, the mean length of sprout on the 60th day was 262.82 ± 16.52 mm, 202.11 ± 14.32 mm, and 255.76 ± 19.73 mm, respectively. The analysis of variance indicated that coppicing height and the initial seedling vigor had significant effects on sprout length (*p* < 0.05). There was also a significant interaction effect of coppicing height and initial seedling vigor on sprout length (*p* < 0.001). On longer coppices, the sprouts grew faster from vigorous seedlings at transplanting (IBSD > 30 mm); while on shorter coppices, sprouts grew faster from thinner seedlings (IBSD ≤ 25 mm).

#### 2.2.2. Growth Rate of Main Stem in Diameter

**Figure 5:**
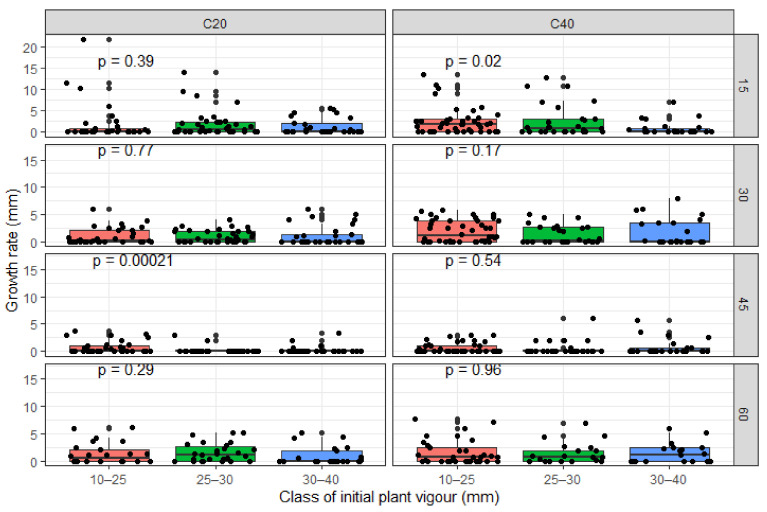
Mean stem growth rate in diameter per coppicing regime and per initial plant vigor over 60 days after pruning. Coppicing regimes: C20 = coppicing at 20 cm above ground; C40 = coppicing at 40 cm above ground. Initial vigor classes: ISBD1 = 10–25 mm; ISBD2 = 20–30 mm, ISBD3 = 30–40 mm. p-values indicated significance level of difference in growth rate among treatments. *p* > 0.05: No difference; *p* < 0.05: significant difference; *p* < 0.001: highly significant difference.

Data indicated that stem growth rate (basal stem diameter) was higher in high coppices (Figure 5). On average, the basal stem diameter growth increment was 1.10 ± 0.11 mm in short coppices and 1.47 ± 0.12 mm in high coppices. It was also noticed that on average, the basal stem diameter growth speed was higher in the first 15 days after pruning, and then gradually slowed down up to 45 days after pruning (DAP), which was seen in all treatments. From 60 DAP, a new increase in the diameter growth rate was recorded. The analysis of variance indicated that the stem growth rate significantly increased with increasing coppicing height (*p* = 0.0396), and with initial seedling vigor at transplanting (*p* = 0.0415). However, no significant interaction effect of the applied management treatments was observed (*p* > 0.05). Higher coppices (pollarding at 40 cm above ground) grew faster in basal diameter compared with shorter coppices (pollarding at 20 cm above ground). Diameter growth rate was higher in the trees that grew from thinner seedlings, compared with those from vigorous seedlings.

#### 2.2.3. Biomass Yield

**Figure 6:**
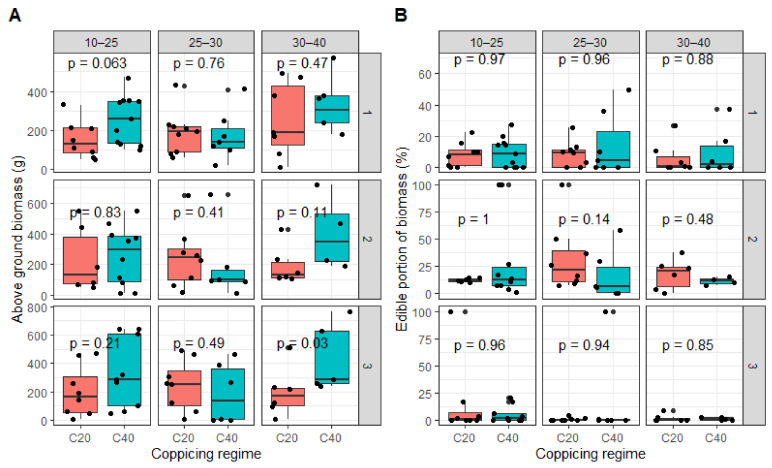
(**A**) Above ground biomass, and (**B**) edible portion of biomass per coppicing regime and per initial plant vigor, over three harvests. Coppicing regimes: C20 = coppicing at 20 cm above ground; C40 = coppicing at 40 cm above ground. Initial vigor classes: ISBD1 = 10–25 mm; ISBD2 = 20–30 mm, ISBD3 = 30–40 mm. p-values indicated significance level of difference in above ground biomass production among treatments. *p* > 0.05: No difference; *p* < 0.05: significant difference.

The above ground biomass included the edible fresh and tender leafy vegetables, and the non-edible lignified twigs and old hard leaves. The results indicated that the above ground biomass varied from 10 to 570 g, 0 to 720 g, and 0 to 765 g in the first, second, and third harvests, respectively (Figure 6A). All treatments together, the edible portion varied from 9.06% to 21.82% of the above ground biomass on average (Figure 6B).

Data showed that *Vitex doniana* leaves contained on average 32% dry matter per 100 g of edible portion. Overall, the dry matter of edible portion varied widely, from 0.98 g to 12.98 g per plant, equivalent to 2.45 kg to 32.45 kg dry matter per hectare and per harvest. The highest edible biomass yield was obtained in the first harvest and decreased over time to become insignificant at the third harvest (Figure 6B). Compared with the first harvest, biomass yield in short coppices was 8.8% less in the second and 127% less in the third harvest. However, the proportion of edible biomass was slightly higher in the second harvest. It is worth indicating that the last harvest coincided with the period of the lowest rainfall in the year (January). Nonetheless, the analysis of variance indicated there is no significant effect of coppicing regime and initial seedling vigor at transplanting on biomass yield (*p* > 0.05). The interaction of the applied treatments was also not statistically significant (*p* > 0.05).

### 2.3. Effects of Initial Vigor and Coppicing on Plant Growth in the Medium Term

#### 2.3.1. Stem Basal Diameter

**Figure 7:**
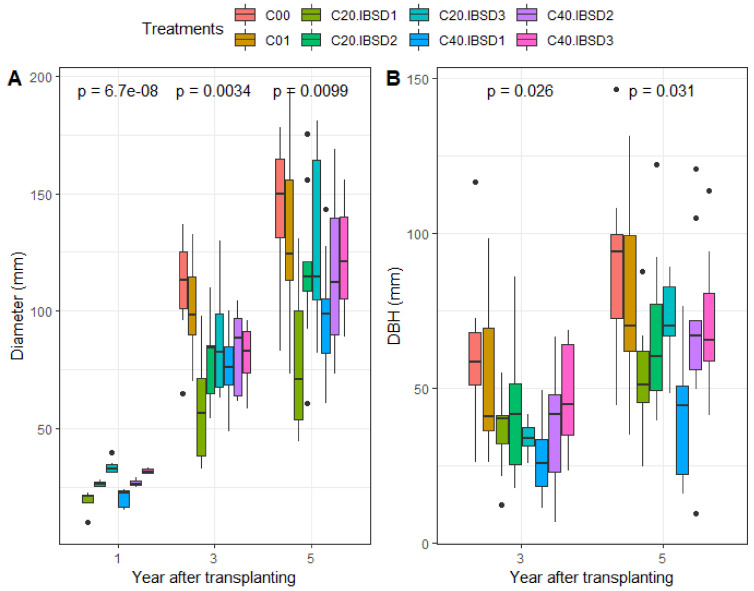
(**A**) Basal stem diameter, and (**B**) diameter at breast height of human (DBH) in three-and five-year old *V. doniana* trees. Coppicing regimes: C00 = Control; C01 = light thinning (i.e., removal of leaves from main stem but leaving crown intact); C20 = coppicing at 20 cm above ground; C40 = coppicing at 40 cm above ground. Initial vigor classes: ISBD1 = 10–5 mm; ISBD2 = 20–30 mm, ISBD3 = 30–40 mm. p-values indicated significance level of difference in diameter / DBH among treatments. *p* < 0.05: significant difference; *p* < 0.01: very significant difference; *p* < 0.001: highly significant difference.

Stem basal diameter of the control trees (C00) was on average 110.5 ± 23.6 mm and 143.3 ± 31.8 mm in the three-year and five-year old trees, respectively. Compared to the control, trees which received light thinning (C01) showed a slightly lower stem growth: 102.1 ± 21.7 mm and 134.7 ± 41.9 mm in three years and five years, respectively. Overall, slower stem diameter growth was observed in all coppiced trees that showed smaller basal stem diameter (Figure 7A). The variance analysis indicated a highly significant effect of coppicing height (*p* < 0.001) and significant effect of the initial seedling vigor (*p* < 0.05) on basal stem diameter after five years. The coppices from vigorous seedlings at transplanting grew faster in stem basal diameter. However, the interaction effect between coppice height and the initial seedling vigor was not significant (*p* > 0.05). It was inferred that coppicing and harvesting of trees reduced tree growth in the stem basal diameter.

#### 2.3.2. Diameter at Breast Height

Overall, the DBH ranged from 0.66 cm to 11.67 cm in three-year old trees, and 0.95 cm to 14.65 cm in five-year old trees. On average, the DBH was higher in the control trees (6.24 ± 2.60 cm and 9.10 ± 3.01 cm in three- and five-year old trees, respectively) (Figure 7B). Smaller mean DBH was recorded in individuals growing from initially thinner seedlings at transplanting and undergoing short coppices (27.45 ± 12.87 mm and 41.51 ± 23.05 mm in three-year and five-year, respectively) compared to those growing from vigorous seedlings at transplanting and undergoing higher coppices (42.74 ± 23.91 mm and 71.46 ± 15.79 mm for three and five-years, respectively). The generalized mixed model indicated that coppicing significantly reduced DBH, and shorter coppices showed smaller DBH (*p* < 0.001). The initial seedling vigor also significantly affected DBH in three- and five-year old trees (*p* < 0.05). It was also found that the DBH of trees that received light thinning (C01) was not significantly different from the control (C00) (*p* > 0.05). Thus, harvesting leafy vegetables on *V. doniana* without cutting the stem and branches does not significantly affect wood production, though vegetable yield will be lower. Moreover, there was no significant interaction between coppicing and initial seedling vigor at transplanting on DBH (*p* > 0.05).

#### 2.3.3. Production of Branches

Overall, the number of branches on three-year old *V. doniana* trees varied with the treatments applied (Table 1). On average, the control trees recorded at least ten branches per tree, compared with not more than eight branches in the treated trees. A closer observation indicated that the treated trees had relatively higher number of branches below the coppicing points. The variance analysis indicated a significant difference between the treated and control trees for the number of total branches (*p* < 0.05). There was a significant effect of the coppicing regime, while no significant effect was observed for the initial seedling vigor on branch production in the medium term (Table 2). The interaction effect between the studied factors was not significant (*p* > 0.05).

#### 2.3.4. Production of Root Suckers

The number of root suckers varied between zero and three per tree in three-year old trees. On average, the number of root suckers produced by three-year old trees was higher in the control trees (C00 and C01) compared with those receiving management treatments (Table 1). The same trend was observed in five-year old trees, although the number of root suckers significantly decreased (data not shown). The variance analysis indicated a significant effect of coppicing regime on the number of root suckers produced in three-year old trees (*p* < 0.05), while no significant effect of the initial plant vigor was observed (Table 2). The interaction effect between coppicing and initial seedling vigor was not significant (*p* > 0.05). We concluded that a coppicing regime is a key factor to control suckering in the species.

## 3. Discussion

### 3.1. Growth and Development of V. doniana

Our study represents the first to investigate *V. doniana* growth and development in cultivation. The findings indicate that it is a fast-growing species, with the height of control trees ranging between 2.17 and 5.89 m tall (4.14 m on average) three years after transplanting. Five-year plantation trees reached between 2.72 and 7.73 m tall (5.36 m on average). These values are by far superior to the reported 1.70 m [14], though this latter value likely referred to unattended plants. In this study, the trees were tendered for 12 months with regular weeding, water supply, and protection from bush fires and uncontrolled harvests. This tendering could explain the wide variation in growth parameters between the cultivated and wild plants of *V. doniana*, and also the 100% survival rate compared to 80–90% reported earlier [14]. DBH of control trees ranged between 2.60 and 11.7 cm (average 6.24 cm) and between 4.46 and 14.65 cm (average 9.10 cm) in three- and five-year old trees, respectively. These values of DBH are comparable to that of *Tectona grandis*, another commercial timber species of the Lamiaceae family, where mean quadratic circumference of young plantation trees (below 5 years) was 23.13 ± 0.62 cm (ca. 5.43 cm DBH), in Southern Benin [15]. Most interesting, five-year *V. doniana* trees in the control showed higher DBH compared with the 29.67 ± 1.21 cm circumference (ca. 6.15 cm DBH) of a teak plantation of more than five-year old [15]. Taking into account that *V. doniana* timber also has physical properties comparable to that of *T. grandis* [5], the former represents a potential commercial multi-purpose tree crop.

### 3.2. Initial Seedling Vigor Determined Plant Growth and Yield

Initial seedling vigor is critical for the success of any plantation project. Although 100% of the transplanted seedlings survived, the findings indicated that more vigorous seedlings grew faster in height, with higher sprouting capacity after coppicing. This confirms hypothesis 1, that the initial seedling vigor at transplanting determined sprouting intensity in *V. doniana.* Empirically, there is a positive correlation between seed mass and seedling vigor with a positive effect on plant growth and biomass yield both in herbaceous and tree crops [12]. In our experiment, sprouts regenerating from more vigorous plants grew faster in height and diameter compared with thinner trees. These results could be explained by more availability of reserves (water and nutrients) for the sprouts growing from more vigorous stands, compared with thinner ones. On the other hand, vigorous stands have a more vigorous rooting system [16,17], thus allowing for higher capacity to capture and use water and nutrient reserves from the soil. In addition, in light of a previously established relationship between leaf production and plant diameter and height [18], vigorous plants also had a higher number of leaves, thus a higher photosynthetic activity, which is ideal for better plant growth. However, the initial seedling vigor at transplanting did not significantly affect suckering capacity in the species. In fact, suckering in plant species is under the control of physiological mechanisms, which involve plant hormones [19].

### 3.3. Positive Response of V. doniana to Coppicing

Our results indicated that the species responds positively to short rotation coppices and a coppicing regime is crucial for sprouting intensity. The short coppices on initially vigorous seedlings produced more sprouts than higher ones, and confirmed hypothesis 2 that coppicing at lower height promotes regrowth and biomass yield in the species. This response is partly explained by the fact that short coppices are more likely to be connected with the roots, which increases the availability of water and metabolites that are needed in sprouting-buds [11,20,21].

However, shorter coppicing could also have adverse effects on sprout growth. Partial or total removal of the above-ground biomass of trees can result in the interruption of root growth or partial death in the root system [22,23], and subsequently reduce plant growth. Leaf removal is assumed to be detrimental to root formation because they supply root promoters such as indole 3-acetic acid (IAA), auxin and other rooting cofactors, vitamins, carbohydrates, organic nitrogen [24,25]. Thus, the greater the leaf area, the better the root and shoot growth. This explains that leafy stands and cuttings are likely to promote more roots and shoot growth than leafless stands. In addition, high (leafy) coppices had a larger number of buds and thus a stronger sink activity, which would therefore increase the metabolic activity and health of leaves. Leaves will then continue to supply photosynthates and rooting cofactors [21]. Our findings are consistent with this observation where (leafless) 20 cm coppices, although showing higher sprouting capacity, did not result in better growth compared with higher (leafier) coppices. In our experiment, coppicing likely induced a slow growth in root biomass, and with more severe effect on the development of the root system of shorter coppices. This is in agreement with theories on balanced growth in plants [26]. This means that coppicing modifies the shoot–root ratio and the response of the affected trees is either the quick activation of sprout-producing buds to trigger the photosynthetic activity at the expense of other organs such as roots (case of leafless short coppices) or, when at all possible, to prioritize the supply of rooting cofactors and carbohydrates to sustain root function (the case of leafier high coppices). The effect of coppicing height on sprouting is also partially attributed to the location of sprout-producing buds on the main stem [11]. It was observed in *V. doniana* that the number of buds increased with the stem segment considered. The last bottom segment of stem (close to ground) had more sprout-producing buds than the upper segments.

In the majority of cases reported in the literature, biomass yield increased with increased coppicing and pruning height [13,27,28]. Nonetheless, although rare, the opposite trend was also reported in some species (e.g., willow (*Salix sp.*) and birch (*Betula pubescens* Ehrh.) [11]). Conceptually, biomass yield is known to be affected by the amount of available carbohydrates for the growing trees [22,29]. Therefore, the most plausible scenario in our study would be that leafier coppices (here coppices of 40 cm) would produce higher biomass compared with leafless coppices (short coppices of 20 cm). However, the effect of coppicing height on biomass may not be conclusive after only one rotation experiment. It is worth indicating that the data reported in this study represents only one rotation and could explain our finding that there was no significant effect of coppicing height on biomass yield. The season of coppicing also affects biomass production by coppiced trees in many species, with the dormant season being more favorable for the harvest of protein-rich leaves [11]. Decline in biomass yield within a rotation can also be due to the frequency of harvest. High harvesting frequency leads to rapid decrease in biomass production [13,30]. In our study, *V. doniana* was harvested every two months and could explain the rapid decline of biomass yield from one harvest to the other. Although seasonality was not assessed in the current trial, the decline in biomass yield could also be due to the season in which the trees were harvested. In fact, the last harvest happened in the driest month, and reduced water availability could explain the low yield.

### 3.4. Trade-Off between Leaves and Wood Production by Coppiced Plants

The findings indicated that coppicing and subsequent pruning allowed for more sprout development, which can be harvested for vegetables, in the short-, but also in the medium-term. In fact, the coppiced trees continued to produce a relatively higher number of branches at lower heights (below 0.40 m and 1.30 m) compared with the control. This impact of coppicing on the plant’s architecture represents an advantage for horticulture prospects because *V. doniana* is a giant tree when fully developed. In the meantime, it was also observed that the coppiced trees to produce edible leaves had the lowest DBH compared with the control trees. This finding confirms hypothesis 3, that coppicing as cultivation management to produce edible leaves negatively affects wood yield in the species. The drastic reduction of stem growth in those trees intensively harvested for their leaves clearly indicates a trade-off that should be taken into account in the production objectives. Simulation studies using growth models have shown that frequent pruning leads to the depletion of reserve carbohydrates in the stems [29]. Therefore, leaves will be produced at the expense of stem growth. This result implies that wild stands that are intensively harvested in nature are subject to decline [6] due to reduced growth, which will affect their reproductive capacity. However, although coppicing affected the timber stock, the coppiced and frequently pruned trees can still yield a substantial quantity of firewood, which was not measured in the current study.

### 3.5. Implications for Further Research

Our study showed contrasting effects of coppicing height on sprouting intensity and on sprout growth, and could justify the hardly perceptible effect of coppicing height on biomass yield. This is supported by a modification of resource allocation and net photosynthesis in coppiced trees [26]. This should be taken into account in further research aiming at simulating the functional structure of the species as a response to cultivation management, especially to coppicing and pruning frequency because they modify the architecture, leaf area, and consequently the photosynthetic processes and crop yield [31,32].

In addition, further investigation should identify the exact distance from which the inhibitory effect of apical buds is suppressed to allow better sprouting and biomass yield, by exploring between 20 to 130 cm above ground. Further studies are also needed to determine the dynamics of shoot–root ratio in coppiced trees. It is known that coppicing and pruning modify the shoot–root ratio [32]. The physiological response to coppicing is the modification of tree growth to restore this equilibrium between above- and below-ground biomass. This explains our finding that short coppices (higher defoliation) quickly produced a higher number of new sprouts to restore this equilibrium. However, the carbohydrate reserves and photosynthetic capacities of subjects determine the speed at which the coppiced trees will grow.

Economic studies are also required to evaluate the profitability of this potential vegetable crop. As a wild-harvested resource, the majority of farmers will engage in the cultivation of *V. doniana* when there is clear evidence of its profitability. To this end, robust environmental economics and crop yield models would be necessary to determine the profitability of the commercial production of *V. doniana*. Furthermore, comparative studies are desirable to determine the profitability of a dual-purpose (vegetables and wood) *V. doniana* production. In fact, our study revealed a trade-off between leaves and wood production by the managed trees. However, a stock of firewood or construction poles can be obtained between two rotation cycles devoted to leaf production.

Finally, phytochemical studies are required to compare the nutritional quality of the cultivated versus wild-harvested leaves of *V. doniana.* We hypothesized that manuring would increase the growth and yield in the species. However, how this will affect the organoleptic and nutritional qualities of the produce is worth investigating in a formal nutrition and consumer sensory test protocol.

## 4. Materials and Methods

### 4.1. Experimental Site

The experiment was conducted at the Agonkanmey Research Station of the National Agricultural Research Institute of Benin (INRAB) in Abomey-Calavi municipality (6°40’ N; 2°33’ E). The climate is sub-equatorial and the average annual rainfall of the site is 1,200 mm. The climate is characterized by two rainy seasons (one long from April to mid-July, and one short from September to November) and two dry seasons (one long from December to March, and mid-July to September). The temperature ranges from 22.8 °C to 32.4 °C, with an annual average temperature of 27.2 °C. Soil in the experimental site is ferralitic soil. The site falls into the Guinean phytogeographical zone and the natural vegetation has been altered by agriculture.

### 4.2. Plant Material and Experimental Design

Data were collected on a *V. doniana* plantation grown for five years. The trees were obtained from sexually propagated seedlings. We tested the response of the species to harvesting as represented by different coppicing regimes (coppicing at different heights, compared to control plants) on different stem diameter classes. Two coppicing heights (C20 = 20 cm, and C40 = 40 cm above ground) were applied on plants with three initial basal stem diameter classes (IBSD1 = 10–25 mm; IBSD2 = 25–30 mm, and IBSD3 = 30–40 mm). In addition to the six cultivation management treatments, there were two controls; one control (C00) where trees received no treatment and another control (C01) where trees were subjected to light thinning (i.e., defoliated by removing all the leaves from the main stem and branches, but not decapitating the tree). This second control simulates a scenario of harvesting without cutting the stem and branches of the trees. There was a total of eight treatments with eight replicates per treatment, in a randomized complete block design (Table 3). Seedlings were transplanted at 2 m × 2 m spacing (2500 stems ha^−1^), and in staggered rows. Seedlings were transplanted after they were grown for five months in a nursery.

### 4.3. Measurements and Data Analysis

Survival rate of transplanted seedlings, height of saplings (monitored from transplanting up to three months), basal stem diameter of growing trees, number and height of sprouts after each pruning, and the edible and non-edible portions of biomass were monitored on a temporal basis over twelve months to assess the effect of the management practices on biomass production. The edible portion of 100 g of leafy vegetable was calculated using the following formula:

(1) $$EPi\;\left(\%\right)\;=\frac{Weight\;of\;edible\;biomass\;\left(g\right)\;in\;treatment\;i\;\times \;100\;}{Weight\;of\;total\;biomass\;\left(g\right)\;in\;treatment\;i}$$

where *EPi* is the edible portion of biomass in treatment *i*.

Other growth parameters such as basal stem dimeter of growing trees, diameter at breast height (DBH, measured at 1.3 m above ground), number of root suckers, number of branches below the pollarding points of 0.2 m, 0.4 m, and below 1.3 m above ground, were recorded when the plantation was three years and five years old. The survival rates in the short and medium terms (one and five years after transplanting) were determined in all treatments using the following formula:

(2) $$SRti\;\left(\%\right)=\frac{Number\;of\;surviving\;trees\;\times \;100}{Number\;of\;transplanted\;seedlings\;in\;treatment\;i}$$

where *SRti* is the survival rate in treatment *i* at date *t*.

The first harvest was conducted six months after transplanting and subsequently implemented every two months, from September to January. A total of three pruning (harvests) series were performed on the trees in the control and in those that received the management treatments. Six series of measurements (every ten days) of the above-mentioned parameters were recorded on each tree after each harvest. Each series of growth and biomass measurement lasted 60 days. Thus, we collected 18 series (three harvests × six series of measurements) of growth data and three biomass yield data over the 12 months. DBH was measured only on three- and five-year old trees as a proxy to estimate wood production. We used mixed effects models with pseudo-replication to account for temporal autocorrelation across repeated measurements on the same trees, and the maximum likelihood method to test the effects of fixed factors (coppicing height and initial seedling vigor at transplanting) and random effect (date of measurement and harvests) on the different growth and yield parameters (main stem’s basal diameter and DBH, the number and growth of new shoots, the number of branches below the pollarding points and below 1.30 m, and the portion of edible biomass). All statistical analyses were performed using R software version 3.6.2 [33].

## 5. Conclusions

The study investigated the response (sprouting intensity and yield parameters) of *V. doniana* to short rotation coppices. Findings clearly indicated a significant effect of initial plant vigor and coppicing regime on the production and growth of sprouts. Furthermore, there was a very clear trade-off between the production of edible leaves and timber production by the coppiced and periodically pruned trees. Coppicing also reduced the formation of root suckers. We recommend that seedlings of at least 30 mm basal stem diameter be used when establishing *V. doniana* plantations, and the coppicing point should not be lower than 40 cm above ground, in order to maximize sprout growth and biomass yield. Future research should investigate higher coppices, seasonality, planting density, fertilization, and their effects on plant architecture and phytochemical composition, as critical elements in the domestication and production of the species.

## Figures and Tables

**Table 1:** Mean number of branches on three-year old trees.

Treatments	Branches Per Tree	Se	Ci	Suckers Per Tree	Se	Ci
**C00**	10.00	1.87	4.42	0.88	0.48	1.13
**C01**	12.13	3.59	8.50	0.75	0.41	0.97
**C40.IBSD1**	6.45	1.33	2.96	0.00	0.00	0.00
**C40.IBSD2**	7.57	1.32	3.24	0.57	0.37	0.90
**C40.IBSD3**	5.17	1.58	4.06	0.00	0.00	0.00
**C20.IBSD1**	5.75	1.49	3.51	0.13	0.13	0.30
**C20.IBSD2**	8.00	0.96	2.21	0.00	0.00	0.00
**C20.IBSD3**	8.00	1.43	3.50	0.43	0.20	0.49

Note: Se, Standard error; Ci, Confidence interval (95%). Coppicing regimes: C00 = Control; C01 = light thinning (i.e., removal of leaves from main stem but leaving crown intact); C20 = coppicing at 20 cm above ground; C40 = coppicing at 40 cm above ground. Initial vigor classes: ISBD1 = 10–25 mm; ISBD2 = 20–30 mm, ISBD3 = 30–40 mm.

**Table 2:** Output of the mixed effects models for branching and root suckering. Intercept condition = control (C00).

Parameters	Estimate	Se	*t* Value	Pr (>|t|)	Estimate	Se	*t* Value	Pr (>|t|)
	Branching				Suckering			
**(Intercept)**	2.50	0.14	18.04	**0.00**	−0.58	0.34	−1.67	0.10
**C01**	0.07	0.19	0.38	0.70	−0.25	0.52	−0.48	0.63
**Coppicing C40**	−0.45	0.21	−2.20	**0.03**	−2.47	1.09	−2.27	**0.02**
**Coppicing C20**	−0.50	0.24	−2.14	**0.03**	−2.06	1.09	−1.90	0.06
**ISBD 25–30**	0.18	0.23	0.79	0.43	2.01	1.13	1.78	0.08
**ISBD 30–40**	0.02	0.26	0.07	0.94	−16.26	2931.19	−0.01	1.00
**Interaction C20 × ISBD 25–30**	0.14	0.33	0.42	0.67	−18.68	2291.41	−0.01	0.99
**Interaction C20 × ISBD 30–40**	0.30	0.36	0.82	0.41	17.36	2931.19	0.01	1.00

Coppicing regimes: Se, Standard error; *t* Value, Student’s t value. Pr (>|t|), Probability associated with Student’s t value. C01 = light thinning (i.e., removal of leaves from main stem but leaving crown intact); C20 = coppicing at 20 cm above ground; C40 = coppicing at 40 cm above ground. Initial vigor classes: ISBD1 = 10–25 mm; ISBD2 = 20–30 mm, ISBD3 = 30–40 mm.

**Table 3:** Treatments applied to *Vitex doniana* trees in a plantation.

**Controls**	No coppicing (C00) Light thinning: removal of leaves from main stem but leaving crown intact (C01)	C00		
		C01		
**Management treatments**	Coppicing regime	Initial seedling vigor (diameter, mm)		
		ISBD1 (10–25)	ISBD2 (25–30)	ISBD3 (30–40)
	20 cm above ground (C20)	C20.ISBD1	C20.ISBD2	C20.ISBD3
	40 cm above ground (C40)	C40.ISBD1	C40.ISBD2	C40.ISBD3
